# Estimation of phytochemical constituents and in vitro antioxidant potencies of *Brachychiton populneus* (Schott & Endl.) R.Br.

**DOI:** 10.1186/s13065-019-0549-z

**Published:** 2019-03-21

**Authors:** Riffat Batool, Muhammad Rashid Khan, Moniba Sajid, Saima Ali, Zartash Zahra

**Affiliations:** Department of Biochemistry, Faculty of Biological Sciences, Quaid-i-Azam University, Islamabad, Pakistan

**Keywords:** *Brachychiton populneus*, Total phenolics, Total flavonoids, Antioxidant

## Abstract

**Background:**

Plants either in raw form or their isolated bioactive constituents are utilized as complementary and alternative medicine in various disorders. The present study was designed to evaluate chief phytochemical constituents of various fractions of *Brachychiton populneus* leaves and its antioxidative aptitude against free radicals.

**Methods:**

Various fractions of *B*. *populneus* were prepared through solvent–solvent extraction technique based on their polarity and screened for phytochemical classes, total phenolic (TPC), flavonoid (TFC) and total tannin (TTC) content. Antioxidant effects of the extracts were manifested by in vitro multidimensional assays i.e. DPPH, hydroxyl radical scavenging, iron chelating, nitric oxide scavenging, β-carotene bleaching, phosphomolybdenum and reducing power assay.

**Results:**

Qualitative screening of various fractions of *B*. *populneus* ensured the presence of alkaloids, saponins, terpenoids, phenols, tannins, triterpenoids and flavonoids. Quantitative analysis revealed that aqueous fraction (BPA) showed maximum quantity of TPC and TFC followed by BPE and BPB. In terms of IC_50_ values BPA exhibited minimum values in all the in vitro antioxidant assays. However, the phytochemicals and yield did not accumulate in various fractions on polarity.

**Conclusion:**

Our results indicated the presence of various polyphenolics, flavonoids, alkaloids etc. The yield of various fractions and qualitative phytochemical analysis did not correlate with polarity of solvents. Various antioxidant assays exhibited significant (p < 0.05) correlation with TPC and TFC and renders *B*. *populneus* with therapeutic potential against free-radical-associated oxidative damages and this effect was significant with BPA.

**Electronic supplementary material:**

The online version of this article (10.1186/s13065-019-0549-z) contains supplementary material, which is available to authorized users.

## Background

Phytochemical studies are based on exploring plants for their use in the production of novel therapeutic drugs. Phytonutrients have numerous health benefits, for example, they may have antimicrobial, anti-inflammatory, anti-diabetic, cancer preventive and antihypertensive properties [1]. Herbal medicinal plants synthesize vast range of secondary metabolites having therapeutic potential to cope with oxidative stress caused diseases [2]. The antioxidant activity of medicinal plants is primarily because of the occurrence of organic substances. Phytochemicals have revealed substantial impact on several pharmaceutical products defining their therapeutic effect which certainly predicts the specific usage and presentation type [3]. Polyphenols enjoy eminent status these days due to latest outcomes and research concerning their biological activities. They are strong antioxidants i.e. are tremendous free radical foragers and inhibitors of lipid peroxidation. Thus have crucial role from pharmacological and therapeutic point of view. Terpenoids are another important class of phytochemicals that are useful for curing obesity induced metabolic disorders [4]. Awareness of chemical components of plants is vibrant for developing new drug products from medicinal plants. Modern isolation methods, screening of biological activities and pharmacological challenges led to the development of purified drugs [5].

The antioxidative aptitude of the therapeutic plants and their derived compounds is directly correlated with their strength to quench the reactive radicals by donation of electron; ultimately leading to radical chain reaction termination. Antioxidants can be produced inside the body [e.g., superoxide dismutase (SOD), reduced glutathione (GSH) etc.] or taken as dietetic antioxidants [1]. Plants are a good source of dietary (i.e. exogenous) antioxidants. Two-third of the world’s plant species have therapeutic importance, and nearly all of them posses tremendous antioxidant prospective. The curiosity in the exogenous plant antioxidants was first educed by the finding and consequent isolation of ascorbic acid from the plants [6].

Insufficient antioxidant defenses lead to the oxidative stress state during the overwhelming generation of reactive oxygen species (ROS) and reactive nitrogen species (RNS). Among the many devastating conditions, oxidative stress causes damages to the nucleic acid, lipids and proteins. This situation is associated with synthesis of secondary reactive species as a response of oxidation. Such continuous metabolic reactions severely harm the cells inducing various diseases through apoptosis and necrosis. Oxidative dilemma is the root cause of many pathological irregularities of liver, lungs, kidneys, brain and heart [7]. It is suggested by scientific documentation that ROS induced cellular damages can be overcome and neutralized through chemo-deterrence by means of therapeutic herbs and foods. On the basis of past achievements about natural products, a variety of medical vegetation has been appraised in favor of their anti-oxidative potential [8].

*Brachychiton populneus* generally known as the Kurrajong is a member of the family Sterculaceae. It is a small to medium-sized tree up to 20 m in height which generally have a moderately short trunk and a compactly-foliaged pinnacle [9, 10]. The genus is reported for innumerable chemical compounds including alkaloids, flavonoids, terpenes, sterols and coumarins that have antioxidants, antimicrobial and antidiabetic potencial [11]. Due to absence of prior biological investigation, the main purpose of this study was to elucidate the phytochemical constituents on qualitative as well as quantitative basis of the various fractions of *B. populneus* and assessment of its antioxidant potential through direct radical foraging methods.

## Results

### Qualitative phytochemical analysis

Qualitative analysis for various phytochemicals viz. alkaloids, anthocyanins, betacyanins, anthraquinones, coumarins, flavonoids, saponins, tannins, terpenoids, glycosides, phenols, steroids, triterpenoids, proteins, vitamin C, phlobatannins and sterols was carried out for *B. populneus* methanol extract and its derived fractions. Results shown in Table 1 indicated that various chemical classes did not obey the polarity of solvents for resolution. These results confirmed the presence of alkaloids, flavonoids, phenols, terpenoids, triterpenoids, quinones, oils and resins, phlobatannins, vitamin C, proteins and glycosides in all fractions of *B. populneus*. Coumarins and saponins were present in all the fractions except BPH. Anthraquinones were present in BPM, BPE, BPB and BPA. Betacyanins were present in BPM, BPH and BPA while BPM, BPC, BPE and BPB contained anthocyanins. Steroids and phytosteroids were present in all the fractions except BPA. Presence of sterols was recorded in BPM, BPE, BPB and BPA. Further, BPA contained the maximum phytochemical classes while BPH showed the least number of existing phytochemicals.

**Table 1:** Phytochemical analysis of *Brachychiton populneus* leaves methanol extract and derived fractions

Compound class	Extracts/fractions					
	BPM	BPH	BPC	BPE	BPB	BPA
Alkaloids						
Mayer’s test	++	+	+	+++	++	+++
Hager’s test	+++	+	+	+++	+++	+++
Tannins						
FeCl_3_ test	+	+	+	++	++	++
Alkaline reagent test	++	+	+	++	+	++
Phenols	++	+	+	+++	+++	+++
Flavonoids						
Alkaline reagent test	++	+	++	+++	++	+++
FeCl_3_ test	++	+	+	+++	+++	+++
Anthraquinones	+	−	−	+	+	++
Betacyanins	++	+	−	−	−	+
Anthocyanins	+	−	++	+++	+	−
Terpenoides	++	+	++	+++	++	+++
Saponins						
Froth test	+	−	+	++	+	+
Emulsion test	++	−	+	++	++	++
Coumarins	+	−	+	++	+	+
Glycosides	++	+	+	+++	+++	+++
Sterols	+	−	−	+	+	++
Oils and resins	+	+++	+	+	++	+
Quinones	+	+	++	++	++	++
Triterpenoids	++	+++	++	+	+	+
Phlobatannins	++	+++	++	+	+	+
Steroids and phytosteroids	+	++	++	+	+	−
Vitamin C	++	+	++	+++	++	+++
Proteins						
Xanthoproteic test	+	+	+	+++	++	+
Biuret test	++	+	+	++	++	++

(+) present, (−) absent, (++) moderate concentration, (+++) abundant concentration

BPM: *B. populneus* methanol extract; BPH: *B. populneus n*-hexane fraction; BPC; *B. populneus* chloroform fraction; BPE: *B. populneus* ethyl acetate fraction; BPB: *B. populneus* butanol fraction; BPA: *B. populneus* aqueous fraction

### Plant yield and quantitative spectrophotometric phytochemical analysis

**Fig. 1:**
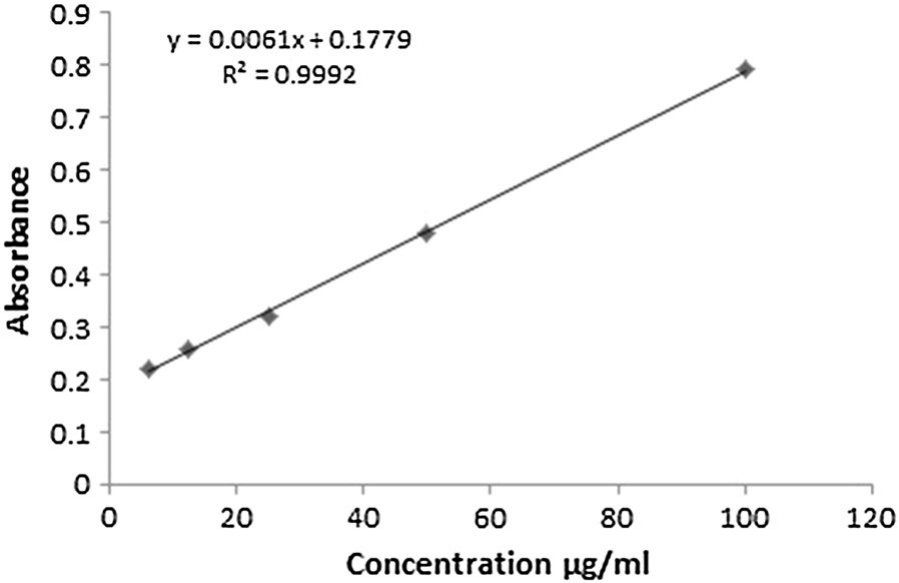
Regression line of gallic acid with total phenolic content

**Fig. 2:**
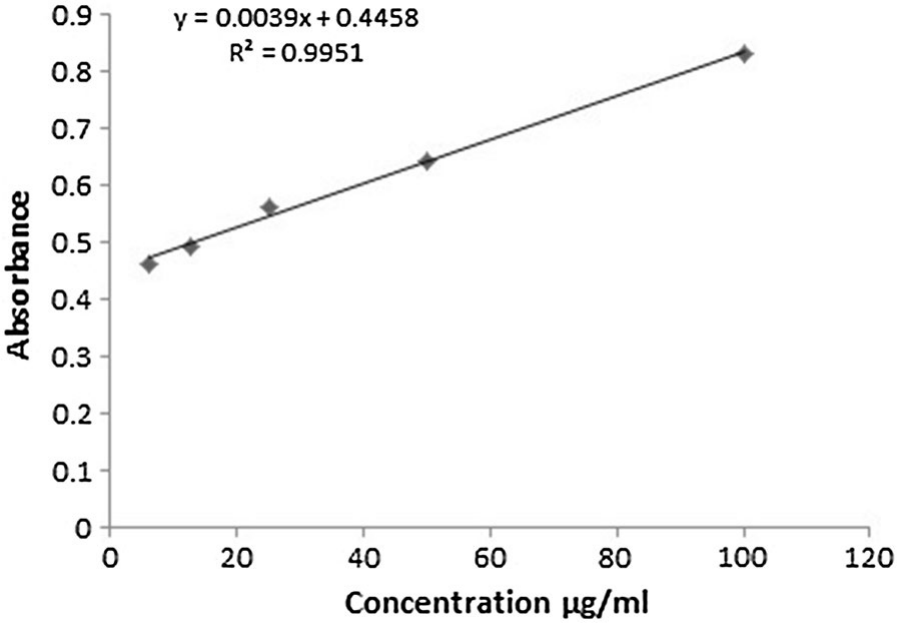
Regression line of rutin with total flavonoid contents

The extraction yield of methanol extract and its various fractions are depicted in Table 2. An amount of 450 g of dry powder of *B. populneus* produced 50 g of crude methanol extract which was progressed with different organic solvents having different polarity index. The yield produced by different solvents during fractionation indicated that it did not follow the polarity of solvents. The maximum yield 17 g was obtained for BPA whereas the yield of other fractions; BPH, BPC, BPE and BPB was found to be 13 g, 7.8 g, 1.5 g and 8.5 g, respectively. On the basis of standard regression lines for gallic acid (Fig. 1) and rutin (Fig. 2), the equivalents of standards were calculated i.e. mg of gallic acid equivalent/g of dry sample (mg GAE/g dry sample) and mg of rutin equivalent/g of dry sample (mg RE/g dry sample) (Table 2). *B. populneus* aqueous fraction (BPA) showed maximum quantity of TFC (126.7 ± 1.15 mg RE/g dry sample) followed by BPE (119.7 ± 2.1 mg RE/g dry sample), BPB (107.7 ± 1.4 mg RE/g dry sample), BPM (99.1 ± 1.05 mg RE/g dry sample), BPC (78.6 ± 1.3 mg RE/g dry sample) and BPH (70.9 ± 2 mg RE/g dry sample) as shown in Table 2. TPC were found to be rich in BPA (189.2 ± 1.6 mg GAE/g dry sample) followed by BPE (174.4 ± 1.2 mg GAE/g dry sample), BPB (162.9 ± 0.9 mg GAE/g dry sample), BPM (156.6 ± 1.17 mg GAE/g dry sample), BPC (149.2 ± 2.1 mg GAE/g dry sample) and BPH (139.4 ± 2 mg GAE/g dry sample). Total tannin content (TTC) was quantified spectrophotometrically as highest in BPE (383.63 ± 0.8 mg of GAE/g dry sample) successively followed by BPA (351.17 ± 0.7 mg of GAE/g dry sample), BPM (280.43 ± 0.5 mg of GAE/g dry sample), BPB (235.3 ± 0.6 mg of GAE/g dry sample) whereas BPC and BPH (72.5 ± 0.65 and 53.8 ± 0.36 mg of GA/g extract) lagged afterwards as shown in Table 2. On the whole the yield accumulated for various fractions did not strictly correlate with the polarity of various solvents used in this study.

**Table 2:** Estimation of plant extraction yield, total phenolics, flavonoids, tannins, antioxidant capacity and reducing power of *Brachychiton populneus* leaves

Sample	Yield (g)^g^	Total phenolic contents expressed as gallic acid equivalents (mg/g of extract)	Total flavonoid contents expressed as rutin equivalents (mg/g of extract)	Total tannin content expressed as gallic acid equivalents (mg/g of extract)	Total antioxidant capacity expressed as ascorbic acid equivalents (mg/g of extract)	Total reducing power expressed as ascorbic acid equivalents (mg/g of extract)
BPM	50	156.6 ± 1.17^d^	99.1 ± 1.05^d^	280.43 ± 0.5^c^	685.4 ± 2.05^c^	885.4 ± 2.06^d^
BPH	13	139.4 ± 2^f^	70.9 ± 2^f^	53.8 ± 0.36^f^	555.6 ± 1.1^e^	822.3 ± 1.8^f^
BPC	7.8	149.2 ± 2.1^e^	78.6 ± 1.3^e^	72.5 ± 0.65^e^	592.5 ± 1.37^d^	850.19 ± 2.6^e^
BPE	1.5	174.4 ± 1.2^b^	119.7 ± 2.1^b^	383.63 ± 0.8^a^	759.03 ± 2.28^b^	956.2 ± 1.71^b^
BPB	8.5	162.9 ± 0.9^c^	107.7 ± 1.4^c^	235.3 ± 0.6^d^	685.4 ± 1.29^c^	933.6 ± 3.04^c^
BPA	17	189.2 ± 1.6^a^	126.7 ± 1.15^a^	351.17 ± 0.7^b^	851.65 ± 2.2^a^	988.34 ± 2.1^a^

BPM: *B. populneus* methanol extract; BPH: *B. populneus n*-hexane fraction; BPC: *B. populneus* chloroform fraction; BPE: *B. populneus* ethyl acetate fraction; BPB: *B. populneus* butanol fraction; BPA: *B. populneus* aqueous fraction

Each value is represented as mean ± SD (n = 3). Means with different superscript (^a−f^) letters in the rows are significantly (p < 0.01) different from one another

^g^Yield of BPM is based on the dry powder; the yield of its fractions is based on the yield of BPM

### Quantitative non-spectrophotometric phytochemical analysis

Various fractions of *B. populneus* were quantified for major phytochemicals including alkaloids, terpenoids, flavonoids and saponins whose presence was observed in qualitative phytochemical analysis directed preliminarily. All results were expressed as percent of yield/g of sample as presented in Table 3. Maximum amount of alkaloids was detected in BPA (18.1 ± 0.27) followed by BPE (16.7 ± 0.55). BPB, BPM, BPC and BPH trailed behind as shown in Table 3. Terpenoids were weighed maximum in BPH (17.2 ± 0.73) sequentially tracked by BPC (12.9 ± 0.85), BPM (10.97 ± 0.35), BPE (9.5 ± 0.21) and BPB (8.7 ± 0.21), while the least terpenoid content was displayed by BPA (6.5 ± 0.55). Flavonoid percentage was detected highest in BPE (15.7 ± 0.2) followed closely by BPA (14.3 ± 0.7). BPB presented flavonoid percentage of 10.8 ± 0.21, whereas BPH showed minimal amount of flavonoids (2.21 ± 0.6). Saponins were quantified as highest in BPM (20.4 ± 0.29) followed by BPB (17.3 ± 0.5), BPA (16.1 ± 0.26), BPE (14.8 ± 0.41) and BPC (12.5 ± 0.72) whereas least in BPH (8.0 ± 0.11) (Table 3).

**Table 3:** Non-spectrophotometric quantitative phytochemical analysis of *B. populneus* and its derived fractions

Plant extracts/fractions	Percentage (%) yield per gram			
	Alkaloids	Flavonoids	Saponins	Terpenoids
BPM	6.93 ± 0.51	5.96 ± 0.33	20.4 ± 0.29	10.97 ± 0.35
BPH	2.3 ± 0.75	2.21 ± 0.6	8.0 ± 0.11	17.2 ± 0.73
BPC	3.8 ± 0.37	7.29 ± 0.52	12.5 ± 0.72	12.9 ± 0.85
BPE	16.7 ± 0.55	15.74 ± 0.2	14.8 ± 0.41	9.5 ± 0.40
BPB	10.2 ± 0.4	10.8 ± 0.21	17.3 ± 0.5	8.7 ± 0.21
BPA	18.1 ± 0.27	14.3 ± 0.7	16.1 ± 0.26	6.5 ± 0.55

Mean ± SD (n = 3)

BPM: *B. populneus* methanol extract; BPH: *B. populneus n*-hexane fraction; BPC: *B. populneus* chloroform fraction; BPE: *B. populneus* ethyl acetate fraction; BPB: *B. populneus* butanol fraction; BPA: *B. populneus* aqueous fraction

### In vitro antioxidant activities

#### DPPH radical scavenging activity

**Fig. 3:**
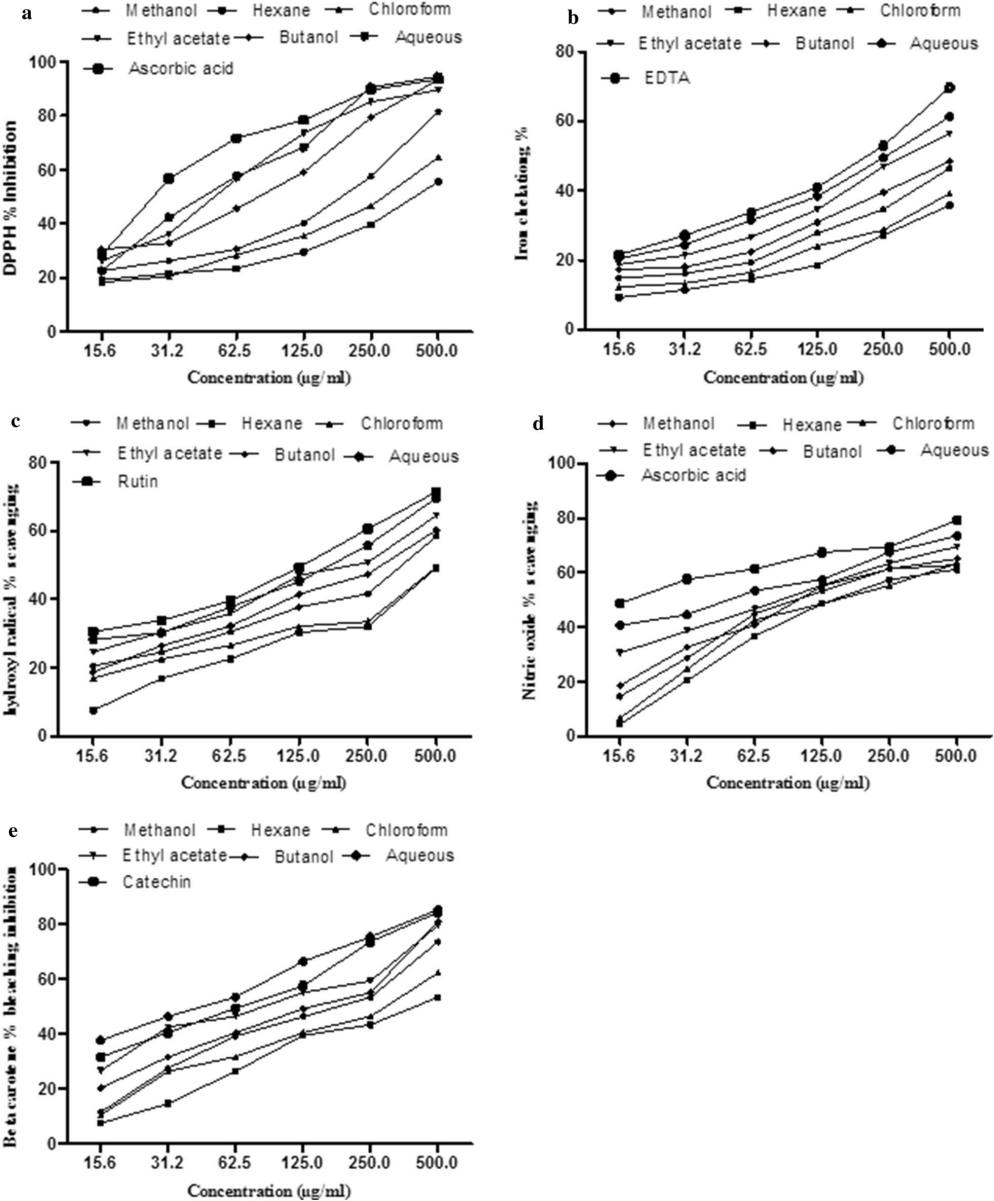
Effect of different concentrations of BPM and its derived fractions on various in vitro antioxidant assays. **a** DPPH percent inhibition, **b** percent of iron chelation, **c** hydroxyl radical percent scavenging, **d** nitric oxide percent scavenging, **e** β-carotene bleaching percent inhibition

The IC_50_ values of DPPH radical scavenging activity of *B. populneus* extract/fractions are shown in Table 4. Best values for IC_50_ were exhibited by BPA (46.51 ± 2.1 µg/ml) followed by BPE (48.32 ± 2.1 µg/ml), BPB (63.38 ± 3.4 µg/ml), BPM (143.7 ± 2.7 µg/ml), BPC (259.6 ± 3.3 µg/ml) and BPH (461.7 ± 1.5 µg/ml). The observed order of IC_50_ of different fractions was BPA < BPE < BPB < BPM < BPC < BPH. The DPPH radical scavenging activity of extract and its various fractions showed significant correlation with TPC (R^2^ = 0.8529**, p < 0.01) and TFC (R^2^ = 0.8567**, p < 0.01) (Table 5). All the fractions showed higher IC_50_ values than ascorbic acid (29.57 ± 1.1 µg/ml). Concentration dependent activity was observed as illustrated in Fig. 3.

**Table 4:** IC_50_ values of different antioxidant activities of BPM and its fractions

Sample	DPPH scavenging	Hydroxyl radical scavenging	Nitric oxide scavenging activity	β-carotene bleaching inhibition activity	Iron chelating activity
	IC_50_ (μg/ml)				
BPM	143.7 ± 2.7^c^	345.6 ± 2.1^c^	130 ± 2.9^c^	148.8 ± 2.3^c^	761.5 ± 1.9^c^
BPH	461.7 ± 1.5^a^	618.3 ± 4.0^b^	180.4 ± 3.3^a^	347.3 ± 3.6^a^	> 1000
BPC	259.6 ± 3.3^b^	764.8 ± 2.5^a^	165.5 ± 2.7^b^	244.8 ± 2.8^b^	> 1000
BPE	48.32 ± 2.1^e^	180.5 ± 3.6^e^	82.7 ± 3.08^d^	77.9 ± 1.5^e^	336.7 ± 1.8^e^
BPB	63.38 ± 3.4^d^	255.0 ± 2.2^d^	121.2 ± 2.5^c^	115.3 ± 2.1^d^	605.4 ± 2.3^d^
BPA	46.51 ± 2.1^e^	144.3 ± 3.2^f^	46.49 ± 2.7^e^	40.04 ± 3.1^g^	249.8 ± 2.8^f^
Rutin	–	110.7 ± 1.7^g^	–	–	–
Ascorbic acid	29.57 ± 1.1^f^	–	16.4 ± 1.6^f^	–	–
Catechin	–	–	–	58.4 ± 2.8^f^	–
EDTA	–	–	–	–	177.2 ± 2.8^g^

Values are presented as mean ± SD (n = 3). Means with different superscript (a–g) letters in the rows are significantly (p < 0.01) different from each other

BPM: *B. populneus* methanol extract; BPH: *B. populneus n*-hexane fraction; BPC: *B. populneus* chloroform fraction; BPE: *B. populneus* ethyl acetate fraction; BPB: *B. populneus* butanol fraction; BPA: *B. populneus* aqueous fraction

**Table 5:** Correlation of IC_50_ values of different antioxidant activities with total phenolic and total flavonoid contents

Antioxidant activity	Correlation R^2^	
	TFC	TPC
DPPH scavenging activity	0.856**	0.852**
Hydroxyl radical scavenging activity	0.888**	0.721*
Iron chelating assay	0.988***	0.872**
Nitric oxide scavenging activity	0.949***	0.988***
β-Carotene bleaching activity	0.956***	0.876**
Total antioxidant activity	0.941***	0.977***
Total reducing power assay	0.978***	0.953**

TFC: total flavonoid content; TPC: total phenolic content

Column with different *, **, *** are significantly different at p < 0.05, p < 0.01 and p < 0.001

#### Hydroxyl radical (•OH) scavenging activity

All the extract/fractions of *B. populneus* scavenged •OH radicals and prevented 2-deoxyribose breakdown in this assay. A concentration-dependent pattern was observed for hydroxyl radical scavenging activity (Fig. 3). Lowest IC_50_ values were shown by BPA and BPE (144.3 ± 3.2 μg/ml and 180.5 ± 3.6 μg/ml) respectively followed by BPB (255.0 ± 2.2 μg/ml), BPM (345.6 ± 2.1 μg/ml) while the highest IC_50_ was observed for BPH and BPC (618.3 ± 4.0 μg/ml and 764.8 ± 2.5 μg/ml) respectively. IC_50_ values of different fractions were significantly different from the used standard rutin (110.7 ± 1.7 μg/ml). Overall pattern of BPA < BPE < BPB < BPM < BPH < BPC was observed (Table 4). A good correlation (R^2^ = 0.7216*, p < 0.01) was observed with TPC as well as (R^2^ = 0.8881**, p <0.01) with TFC (Table 5).

#### Nitric oxide (NO^−^) scavenging activity

In the present study, the lowest IC_50_ value for nitric oxide scavenging activity was observed for BPA (46.49 ± 2.7 μg/ml) and BPE (82.7 ± 3.08 μg/ml) followed by BPB (121.2 ± 2.5 μg/ml), BPM (130.0 ± 2.9 µg/ml), BPC (165.5 ± 2.7 μg/ml) and BPH (180.4 ± 3.3 μg/ml) as compared to standard ascorbic acid (16.4 ± 1.6 µg/ml) as shown in Table 4. The % inhibition pattern is shown in Fig. 3. A highly significant (p < 0.01) correlation of IC_50_ was observed with TPC (R^2^ = 0.988***, p < 0.001) and TFC (R^2^ = 0.9494***, p < 0.001) (Table 5).

#### β-Carotene scavenging activity

The BPA of *B. populneus* showed the lowest IC_50_ value (40.04 ± 3.1 μg/ml) as compared to other fractions viz. BPE (77.9 ± 1.5 μg/ml), BPB (115.3 ± 2.1 μg/ml), BPM (148.8 ± 2.3 μg/ml), BPC (244.8 ± 2.8 μg/ml) and BPH (347.3 ± 3.6 μg/ml). All the fractions showed higher IC_50_ values than catechin (58.4 ± 2.8 μg/ml) while IC_50_ value of BPA was lower than catechin as shown in Table 4. The concentration dependent bleaching power pattern observed is shown in Fig. 3. The IC_50_ values showed significant correlation with both TPC (R^2^ = 0.8764**, p < 0.01) and TFC (R^2^ = 0.9566***, p < 0.001) (Table 5).

#### Iron chelating activity

IC_50_ values for Iron chelating activity of different fractions of *B. populneus* are given in (Table 4). The best IC_50_ value for iron chelating activity was exhibited by BPA (249.8 ± 2.8 µg/ml) followed by BPE (336.7 ± 1.8 µg/ml), BPB (605.5 ± 2.3 µg/ml) and BPM (761.6 ± 1.9 µg/ml) while BPC and BPH showed the higher IC_50_ values (> 1000 µg/ml). IC_50_ value of standard EDTA was 177.2 ± 2.8 µg/ml as shown in Table 3. Significant correlation of IC_50_ (R^2^ = 0.8721**, p < 0.01) was observed with TPC and also with TFC (R^2^ = 0.9888***, p < 0.001) as listed in Table 5. The  % inhibition of iron chelating assay is depicted in Fig. 3.

#### Phosphomolybdenum assay

**Fig. 4:**
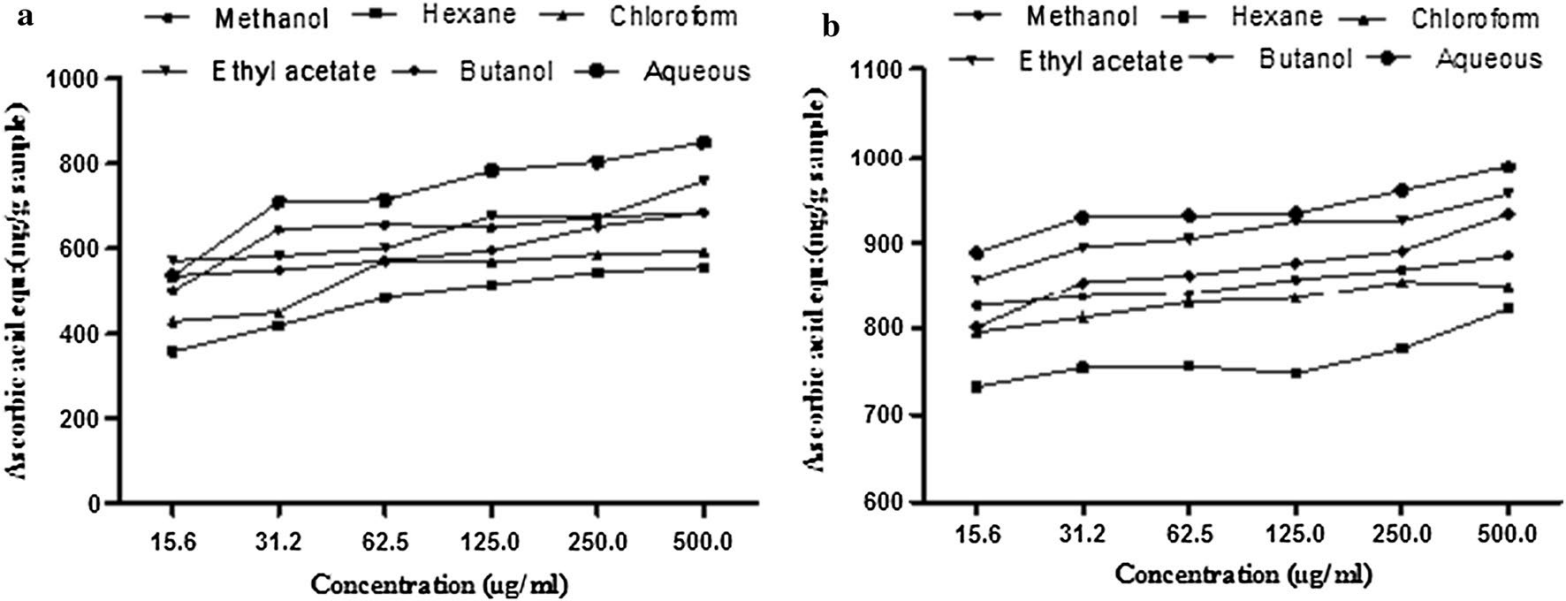
**a** Total antioxidant activity (phosphomolybdate assay), **b** reducing power assay of BPM and its fractions

Total antioxidant capacity of various extract/fractions was determined by phosphomolybdate method and expressed as equivalents of ascorbic acid (mg/g of extract) at 500 μg/ml sample as shown in Fig. 4a. Maximum antioxidant activity was shown by BPA (851.6 ± 2.2 mg ascorbic acid equivalents/g sample) followed by BPE (759.03 ± 2.28 mg ascorbic acid equivalents/g sample), BPB (685.4 ± 0.86 mg ascorbic acid equivalents/g sample), BPM (685.4 ± 205 mg ascorbic acid equivalents/g sample), BPC (592.5 ± 1.37 mg ascorbic acid equivalents/g sample) and BPH (555.6 ± 1.1 mg ascorbic acid equivalents/g sample) and was found to decrease in the order of BPA > BPE > BPB > BPM > BPC > BPH. The assay showed highly significant (p < 0.001) correlation with TPC (R^2^ = 0.9774***) and TFC (R^2^ = 0.9412***) (Table 5).

#### Reducing power activity

*Brachychiton populneus* aqueous fraction showed the highest reducing power (988.34 ± 2.1 mg ascorbic acid equivalent/g sample) measured at 500 μg/ml of extract concentration followed by BPE (956.2 ± 1.71 mg ascorbic acid equivalents/g sample), BPB (933.6 ± 3.04 mg ascorbic acid equivalents/g sample), BPM (885.4 ± 2.06 mg ascorbic acid equivalents/g sample), BPC (850.19 ± 2.6 mg ascorbic acid equivalents/g sample) and BPH (822.3 ± 1.8 mg ascorbic acid equivalents/g sample) as shown in Fig. 4b. There was exhibited a significant correlation (p < 0.001) with both TPC (R^2^ = 0.9534***) and TFC (R^2^ = 0.9783***) (Table 5).

## Discussion

Medicinal plants contain variety of chemical constituents that differ from each other regarding polarity and other chemical properties. Isolation of chemical compounds from plants through solvents of different polarity is frequently practiced in phytochemistry [12]. Depending upon the nature of solvents, different extracts yield differently as described by Shah et al. [13]. So, in the present study, maximum yield was obtained in BPA followed by BPH while BPE produced the minimum yield. Contrary to our results Sahreen et al. [14] reported the lowest yield for hexane fraction in the roots of *Rumex hastatus*.

Therapeutic propensity of the plants can be assessed by performing initial qualitative screening to ensure the presence of phytochemicals. In the conducted study bioactive constituents that confer biologically dynamic nature to the plants were screened and the results confirmed the existence of coumarins, terpenoids, flavonoids, tannins, alkaloids, phenols, saponins, quinones, phytosteroids, triterpenoids, vitamin C, phlobatannins, sterols, glycosides and betacyanin in BPM. In this study the solvents were unable to resolve the presence of phytochemicals on the polarity basis and most of these phytochemicals were in different fractions. Similar results were recorded in other studies [15]. However, *n*-hexane solvent was able to resolve the presence of some phytochemicals and BPH did not constitute anthraquinones, anthocyanins, saponins, coumarins and sterols that were present in BPM. These results suggest the poor solubility of these phytochemicals in *n*-hexane. Apart from this, quantitative spectrophotometric and non-spectrophotometric phytochemical analysis unraveled considerable amount of saponins, alkaloids, flavonoids, phenols, tannins and terpenoids to be present in various fractions of *B. populneus*. These results suggest that ethyl acetate is the solvent of choice for maximum extraction of total phenolic and tannins whereas aqueous fraction accumulated maximum quantity of total flavonoids. The polarity based resolution of chemicals provides a choice for the use of fraction in a particular disorder.

Compounds belonging to the respective groups have been reported to impart various medicinal characteristics to the plants. Tannins are the polyphenolic compounds obtained from plants, have tremendous activity against diarrhea, hemorrhage, virus and hemorrhoids, bacteria, fungi and parasites and also impart anti-cancer and cytotoxic activity [16]. Flavonoids and phenols have vital scavenging role in oxidation, inflammation and cancer [17]. Alkaloids are said to have impact on neurological disorders like Alzheimer’s disease [18] and also have been reported for anticancer activities [19]. Saponins have ability to cope with pests, bacteria and fungi [20]. Presence of these compounds justifies the therapeutic potential of *B. populneus.*

To assess and verify the presence of antioxidant capabilities within plants, a variety of antioxidant assays have been established with varying mechanics and kinetics. These assays enquire the plants in diverse ways before certifying it as an antioxidant. So to appraise the antioxidant capacity of polarity based crude extracts of *B. populneus* viz. BPM, BPH, BPE, BPC, BPB and BPA, a series of antioxidant assays were conducted.

Scavenging activity of *B. populneus* extract for free radicals was estimated by DPPH assay. It is very sensitive and short time assay for checking the antioxidant potential of the plant extracts and compounds. DPPH is one of a stable, nitrogen centered dark violet colored powder which changes from violet to yellow color upon reduction [21]. The change in color extent depends upon scavenging capabilities of antioxidant crude extract or an isolated pure compound as it reduces the DPPH radical by donating hydrogen [22]. Presence of phenolics and flavonoids impart the scavenging capabilities to the plant. Phenolics and flavonoids are greatly extracted in the polar solvents which show good scavenging abilities as they donate electron or hydrogen to stabilize DPPH free radicals. In current study the aqueous fraction of *B. populneus* showed good scavenging ability against DPPH, none of the six fractions showed IC_50_ below ascorbic acid used as standard. Our results are in coherence with Kumaran [23] who reported the antioxidant ability of aqueous extract of Coleus aromaticus. The results stated in the present study showed a significant correlation with both TPC and TFC. The substantial correlation of IC_50_ values with TPC and TFC might be ascribed by the presence of flavonoids and other active polyphenols.

Hydroxyl radical is a potent reactive species which cause severe pathogenies to cell membrane phospholipids and react with poly unsaturated fatty acid. It is very toxic and short lived free radical which initiate chain reaction that damages the cellular integrity [24]. In the present study, the evidence of •OH scavenging activity was estimated through the deoxyribose system. Hydrogen peroxide (H_2_O_2_) reacts with ferrous, generating •OH that react with deoxyribose producing red color. Scavenging activity of •OH is directly proportional to the antioxidant activity of the fraction [25]. In the current study, BPA showed the best activity with lowest IC_50_ value followed by BPE < BPB < BPM < BPC < BPH, compared to the standard used. A significant correlation was observed with TPC as well as with TFC. This shows that *B. populneus* has active components to scavenge hydroxyl radical. Our results conformed with Majid et al. [26] who reported high activity in ethyl acetate and ethyl acetate + water extract.

The principal behind the Iron chelating assay was to decolorize the iron–ferrozine complex by the scavenger’s ability or plant extract ability. The water soluble colored complex is formed by the reaction of Fe(II) with ferrozine. The complex of iron–ferrozine was obstructed by the scavenging constituents that chelates with Fe(II) thus reducing the color intensity of the solution [27]. In the present study, BPA showed the lowest IC_50_ value amongst the entire fractions in comparison to EDTA used as standard. Significant correlation was observed with TPC and TFC.

Griess reagent can be used to estimate the nitric oxide activity. In Griess reagent the compound sodium nitroprusside is decomposed at pH 7.2 producing NO^−^ in aqueous solution. In the presence of oxygen, NO^−^ reacts and produces nitrate and nitrite. Nitrite ion production was hindered by entities having scavenging abilities by consuming the available oxygen [28]. In our current study, BPA and BPE showed relatively good results as compared to rest of the fractions and a significant correlation of IC_50_ values was observed both with TPC and TFC. This is owing to the fact that BPA and BPE have numerous bioactive polyphenols and other phenolic composites which have robust potential to scavenge NO^−^ radicals that account for austere oxidative stress. The research of Duenas et al. [29] and Kilani et al. [30] is in agreement to our findings.

β-carotene bleaching assay was used to estimate the plant antioxidant potential. The principal behind this activity is based on linoleic acid oxidation which is caused by the formation of a complex with β-carotene. Linoleic acid hydroperoxides on reaction with β-carotene bleaches its color and the bright yellow color of the reaction mixture is reduced to light milky color. An antioxidant which is present in the reaction assortment acts on linoleic acid free radicals and releases β-carotene from the complex. This results in the restoration of the yellow color of the solution. The brighter color of the solution shows the stronger antioxidant present in the reaction while absorbance of reaction rapidly decreased in samples without antioxidants [31]. BPA fraction in our study showed the lowest IC_50_ value and a strong correlation with both TPC and TFC. Triantaphyllou [32] also reported promising β-carotene bleaching antioxidant activity of aqueous extracts of the herbs of the family lamiaceae.

Phosphomolybdate is another important in vitro antioxidant assay to access the total antioxidant capacity of the plant extract. The assay principal follows the conversion of Mo (VI) to Mo (V) by extract or the compound which possess antioxidant potential resulting in green phosphate Mo (V). The electron/hydrogen donating pattern of antioxidants depends upon its structure and series of redox reactions occurring in the activity [33]. Our findings showed that aqueous fraction of *B. populneus* has good antioxidant potential due to presence of flavonoid and phenolic contents. Phosphomolybdenum assay showed significant correlation with total flavonoid contents as well as total phenolic contents. Jan et al. [34] also reported the best phosphomolybdenum activity of aqueous extract and a significant correlation with TPC and TFC.

Reducing power of *B. populneus* was assessed by using the potassium ferricyanide reduction method. An antioxidant compound in the test sample causes conversion of iron (Fe^+3^) to ferrous (Fe^+2^) by donating hydrogen and the yellow color of the reaction mixture changes to green. The intense green color in the assay shows the strong antioxidant capacity of the sample which has reducing power [35]. BPA fraction showed the highest value of reducing power when compared with ascorbic acid followed by BPE < BPB < BPM < BPC < BPH at 500 μg/ml. The assay results showed significant correlation with both TPC and TFC. Our study has been supported by the report of Sahreen et al. [17] who reported best reducing power activity of methanol extract of *Rumex hastatus* after butanol.

Use of BPM in carbon tetrachloride intoxicated rats down regulated the expression of genes associated with endoplasmic reticulum oxidative stress and inflammatory pathways in liver [36]. The phytochemicals present in *B. populneus* might ameliorate the oxidative stress by direct scavenging of radicals and/or regulating the expression of genes. The antioxidant effects reported during this study suggest the therapeutic use of *B. populneus* in oxidative stress associated disorders.

## Experimental

### Plant collection

Plant collection was done from Quaid-i-Azam University, Islamabad in January–February 2017. The plant was identified by its native name and then confirmed by senior plant taxonomist; Syed Afzal Shah, Department of Plant Sciences, Quaid-i-Azam University, Islamabad. Voucher specimen (036245) was deposited at the Pakistan Herbarium, Quaid-i-Azam University, Islamabad.

### Preparation of extract

The aerial parts of the plant were washed away to remove dust particles and dried under shade for few weeks. The fully dried plant material was then ground to powder and sieved through 60-mesh topology Willy Mill to get fine powder of same particle size. Extraction was carried out by mixing 1.2 kg of plant aerial part powder with 3 l of commercial methanol at 25 °C for 48 h. Filtration was performed by using Whatman No.1 filter paper. The filtrate was further processed in rotary vacuum evaporator for evaporation and obtained the methanol extract (BPM). Distilled water was used to suspend a part of BPM and then it was passed to liquid–liquid partition. The solvents were used in order of *n*-hexane (non-polar), chloroform, ethyl acetate (polar solvent), butanol (polar solvent). Fractions of these solvents were separated accordingly and named as BPH (hexane fraction), BPC (chloroform fraction), BPE (ethyl acetate fraction) and BPB (butanol fraction). The residue after last fraction was also collected and termed aqueous fraction and abbreviated as BPA. Each fraction was dried, weighed and stored for further pharmacological observations.

### Qualitative phytochemical analysis

Qualitative screening of *B. populneus* methanol extract along with its fractions was performed to identify the active phytochemicals like flavonoids, phenols, tannins, alkaloids, saponins, terpenoids, coumarins, anthocyanins and anthraquinones.

#### Assessment of phenols

For qualitative assessment methodology of Harborne [37] was followed. An amount of 1 mg of each sample was taken and 2 ml of distilled water and 10% ferric chloride was added in it. The confirmation sign of phenols presence was formation of green or blue color.

#### Assessment of flavonoids

*Alkaline reagent test* This test was performed by following the protocol of Trease and Evans [38]. Each sample (1 mg) was added in 1 ml of 2 N sodium hydroxide. The confirmation sign of flavonoids presence was formation of yellow color.

*FeCl*_*3*_ *test* Few drops of FeCl_3_ solution were added in 1 ml of each extract. Existence of flavonoids was indicated by formation of blackish red precipitate [39].

#### Assessment of coumarins

Each sample (1 mg) was endorsed to react with 1 ml sodium hydroxide (10%). Formation of yellow color in test sample was an indication of the presence of coumarins [37].

#### Assessment of saponins

*Froth formation with distilled water* Each sample (2 mg) was mixed with 2 ml of distilled water in the test tube. After this accumulation, the test sample was mixed vigorously for almost 15 min. The formation of a soapy layer indicated the presence of saponins in test samples [37].

*Emulsion test with olive oil* A volume of 1 ml of each sample was poured in test tubes followed by addition of 5–6 drops of olive oil and shaken vigorously to form a stable froth. Formation of an emulsion was the confirmatory sign of saponin presence [39].

#### Assessment of tannins

*FeCl*_*3*_ *test* To 1 mg of each sample, 2 ml of 5% ferric chloride was added. Appearance of greenish black or dark blue color was the indication of tannins presence [38].

*Alkaline reagent test* A volume of 2 ml of 1 N NaOH solution was added in 2 ml of each plant extract. Appearance of yellow to red color showed the presence of tannins [39].

#### Assessment of terpenoids

Each sample (0.5 mg) was taken in the test tube and 2 ml of each chloroform and concentrated sulphuric acid was added to plant samples. Presence of terpenoids was indicated by the formation of brown layer in the middle of other two layers [38].

#### Assessment of anthraquinones

To 1 mg of each sample, hydrochloric acid diluted to 2% was added. The appearance of red color was the confirmatory sign of anthraquinone presence [37].

#### Assessment of anthocyanin and betacyanins

Each sample (1 mg) was taken in the test tube and followed by the addition of 2 ml of 1 N sodium hydroxide. The test sample was boiled at 100 °C for about 10 min. Anthocyanin presence was indicated by the formation of bluish green color while yellow color formation was indicative of betacyanin presence [38].

#### Assessment of alkaloids

*Mayer’s test* Each sample (2 ml) was allowed to react with conc. HCl and a special reagent named Mayer’s reagent. Formation of white precipitates or appearance of green color was indication of alkaloids presence [38].

*Hager’s test* Few drops of Hager’s (Saturated picric acid solution) reagent were added to 2 ml of each plant extract. Formation of bright yellow precipitates specified the manifestation of alkaloids [39].

#### Assessment of glycosides

*Keller Killanis’ test* To 1 ml of each plant extract, 1 ml glacial acetic acid was added and left to cool down. After cooling two drops of FeCl_3_ were added and 2 ml of concentrated H_2_SO_4_ along the walls of test tube was dispensed carefully. Development of reddish brown colored ring at the intersection of two layers indicated the presence of glycosides [39].

#### Assessment of sterols

*Salkowski test* To 2 ml of each of the plant extracts, 5 ml of chloroform was added and then 1 ml concentrated H_2_SO_4_ was carefully dispensed along the walls of the tube. The appearance of reddish color in the lower layer indicated the existence of sterols [39].

#### Assessment of vitamin C

*DNPH test* Dinitrophenyl hydrazine was dissolved in concentrated sulphuric acid and allowed to react with 1 ml of plant sample. Appearance of yellow precipitates indicated the presence of vitamin C in test samples.

#### Assessment of proteins

*Xanthoproteic test* According to this procedure, 1 ml of each plant sample was treated with few drops of conc. nitric acid. Presence of proteins in test samples was indicated by the formation of yellow color.

*Biuret test* An amount of 0.5 mg of each plant test solution was taken and equal volume of sodium hydroxide solution (40%) was added to it. After that few drops of 1% CuSO_4_ solution was added. Appearance of violet color in test samples manifested protein presence.

#### Assessment of steroids and phytosteroids

A volume of 1 ml of chloroform and few drops of concentrated sulphuric acid were added to 1 ml of plant test sample. Formation of brown-colored ring indicated steroids presence whereas appearance of bluish-brown colored ring marked the presence of phytosteroids in the test samples.

#### Assessment of phlobotannins

To 1 ml of each plant sample few drops of 10% ammonia solution were added. Formation of pink-colored precipitates showed the existence of phlobatannins in samples.

#### Assessment of triterpenoids

A volume of 1 ml of Libermann-Buchard Reagent (conc. H_2_SO_4_ + acetic anhydride) was added in 1.5 ml plant test samples. Triterpenoids were determined by the appearance of bluish-green color in the test samples.

#### Assessment of quinones

A volume of 1 ml of each plant sample was allowed to react with 1 ml concentrated sulphuric acid. Appearance of red color manifested the occurrence of quinones.

#### Assessment of oils and resins

*Filter paper test* Each plant sample was applied on filter-paper and checked for the establishment of transparent appearance which was a positive sign for the presence of oils and resins in respective test samples.

### Quantitative spectrophotometric phytochemical analysis

Various fractions of *B. populneus* were evaluated spectrophotometrically, employing standardized procedures for the quantification of chief phytochemical constituents including phenols, flavonoids, and tannins.

#### Total phenolic content (TPC)

Determination of total phenolic content was done by spectrophotometer [40]. A volume of 1 ml of each sample was mixed with 2 ml of Phenol Folin–Ciocalteu mixture following 9 ml of pure deionized water in a volumetric bottle having capacity up to 25 ml. After shaking, 10 ml of 7% Na_2_CO_3_ was added. Vigorous stirring was practiced following instant dilution of the final mixture with pure deionized water making final volume up to 25 ml. After keeping the final mixture at 23 °C for at least 90 min, the optical density was checked at wavelength of 750 nm. The whole assay was repeated thrice for ensuring accuracy against the standard gallic acid. TPC was expressed as mg GAE (gallic acid equivalents)/gram dry weight extract/fraction.

#### Total flavonoid contents (TFC)

The spectrophotometric technique is the easiest and affordable technique for finding out the flavonoid contents within a plant [41]. The reaction mixture was made in a test tube by the scheduled addition of 0.3 ml plant sample, 0.15 ml of NaNO_2_ (0.5 mol/l) along with 0.3 M AlCl_3_·6H_2_O and 3.4 ml methanol (30%). The assortment was kept for 5 min and then 1 ml of 1 M NaOH was mixed in it. At 506 nm wavelength the optical density of the reaction mixture was detected using rutin as standard for comparison using concentrations 0–100 mg/ml.

#### Total tannin content (TTC)

Procedure of Van Buren and Robinson [42], was followed for the quantification of tannin content with slight modifications. According to this procedure, 500 mg plant sample was soaked in 50 ml distilled water. The sample was placed on mechanical shaker for 1 h and then filtered. The filtrate was made up to the mark in volumetric flask (50 ml). A volume of 2 ml of FeCl_3_ (0.1 M) and potassium ferricyanide (0.008 M) prepared in HCl (0.1 N) was mixed with 5 ml of the above filtrate. The absorbance was recorded at 200 nm via spectrophotometer against standard curve of gallic acid and the results were quantified as mg of gallic acid equivalents (GAE)/gram of dry plant extract [43].

### Quantitative non-spectrophotometric phytochemical analysis

*Brachychiton populneus* fractions were quantified employing standardized non-spectrophotometric procedures for the presence of alkaloids, terpenoids, flavonoids and saponins.

#### Quantification of alkaloids

Alkaloids were quantified by following the procedure of [37]. A volume of 7.5 ml of 10% acetic acid prepared in ethanol was added to 10 mg of each plant sample. The sample assortment was covered and endorsed to stand for 4 h time interval. The mixture was then filtered and the subsequent filtrate was concentrated on a water-bath to reduce its volume up to one-fourth of its original volume. The extract sample was then finally precipitated by adding conc. NH_4_OH drop wise. The solution was endorsed to settle and the precipitates were collected after filtration. The residue obtained after washing with dilute NH_4_OH was completely dried and finally weighed to calculate the alkaloid percentage in respective plant samples.

#### Quantification of flavonoids

Flavonoid content was quantified by following the methodology of Krishnaiah et al. [44]. Plant sample (100 mg) was extracted repeatedly with 80% aqueous methanol (10 ml) at room temperature followed by filtration through Whatman-42 filter paper (125 mm). The filtrate was left for complete evaporation in water-bath after transferring into a crucible for complete dryness. The sample was weighed after constant weight obtained.

#### Quantification of saponins

Saponins were quantified following the methodology of Obadoni and Ochuko [45]. An amount of 100 mg of each plant sample was dispersed in 15 ml of aqueous ethanol (20%). The suspension was heated on water bath at 55 °C for 4 h with constant stirring. The mixture was filtered followed by re-extraction with another 15 ml of aqueous ethanol (20%). Both the extracts were combined and concentrated to 4 ml on water-bath at 90 °C. Then 2 ml of di-ethyl ether was added to the concentrate in a separating funnel. Aqueous layer was collected whereas ether layer formed was discarded. The sample was purified by repeating the above process. Finally 5 ml of *n*-butanol was added and *n*-butanol combined extracts were washed twice with 1 ml of 5% aqueous NaCl. The remaining solution was completely evaporated on water-bath. A constant weight of the sample was obtained after placing it in oven and saponin content was quantified as %age yield of plant sample.

#### Quantification of terpenoids

Plant sample (100 mg) was soaked in alcohol and placed for 24 h at room temperature. Next day the extract sample was and the filtrate was thoroughly extracted with petroleum ether. The ether extract obtained was treated as the total terpenoids in the sample [46].

### Antioxidant capacity determination assays

For antioxidant potential determination seven different assays were performed to assess the antioxidant prospective against various free radicals and by different mechanisms of action. Extracts, fractions and positive standards (ascorbic acid, Rutin, catechin and gallic acid) 1 mg were liquefied in 1 ml analytical methanol or DMSO. These solutions were further serially diluted to 1000, 500, 250, 125, 62, 31.25, 15.62 µg/ml. In all assays, same dilutions of samples and standards were used; while standards were altered according to the requirement of assay.

#### DPPH (1, 1-diphenyl-2-picryl-hydrazyl) radical scavenging assay

The DPPH foraging competencies of damaging effects of the free radicals were evaluated by following the methodology of Brand-Williams et al. [47] with slight modifications. The stock solution of DPPH was prepared by dissolving 0.24 g of it in 100 ml of methanol and kept at 20 °C for further use. The stock solution was further diluted with methanol to optimize its absorbance (0.908 ± 0.02) at 517 nm. Now 100 µl of plant samples was mixed with 900 µl of DPPH aliquot and incubated for 15 min at room temperature in dark. Optical density was checked at wavelength of 517 nm by running Ascorbic acid as standard. Antioxidant capacity was determined by following Eq. 1:

1 $$ {\text{Free radical scavenging activity }}\left( {\text{\%}} \right) = \left( {\frac{{{\text{Control absorbance}} - {\text{Sample absorbance}}}}{\text{Control absorbance}}} \right) \times 100 $$

#### Hydroxyl radical scavenging assay

Hydroxyl free radicals scavenging potential of plant extracts was assessed by using the methodology accomplished by Halliwell and Gutteridge [48]. This technique involves mixing of 500 µl of 2.8 mM 2-deoxyribose, being prepared in phosphate buffer (50 mM) maintaining its pH 7.4, EDTA 0.1 M, 200 µl of 100 mM ferric chloride, 100 µl of 200 mM H_2_O_2_ and 100 µl of each plant sample in the reaction recipe. The reaction was initiated by the addition of 100 µl of 300 mM ascorbic acid and incubated at 37 °C for 1 h. After this 1 ml of 2.8% TCA and 1 ml of TBA (1% weight by volume) prepared in 50 mM NaOH were added to the reaction mixture. This whole recipe was heat treated for 15 min in water bath and then placed for cooling. Optical density was recorded at 532 nm. The hydroxyl radical scavenging activity was analyzed by following formula:

$$ {\text{Free radical scavenging activity }}\left( {\text{\%}} \right) = \left( {\frac{{1 - {\text{Sample absorbance}}}}{\text{Control absorbance}}} \right) \times 100 $$

#### Nitric oxide scavenging assay

Bhaskar and Balakrishnan [49] developed the methodology using Griess reagent to assess the antioxidant potential of plant samples. Equimolar quantity of napthylenediamine (0.1%) in distilled water and sulphanilamide (1%) in phosphoric acid (5%) was added to prepare griess reagent. 100 μl of 10 mM sodium nitroprusside being prepared in saline phosphate buffer was added to 100 μl of each plant sample. Then 1 ml of griess reagent was added to, reaction mixture, incubated for 3 h and analyzed spectrophotometrically at 546 nm by using ascorbate as a positive control. The percentage inhibition of nitric oxide radical formation was determined by following Eq. 1.

#### Chelating power assay

The iron (II) binding capability at multiple sites confers the antioxidant potential of plant samples following the methodology of Dastmalchi et al. [50]. A volume of 200 µl of plant sample was taken as plant aliquot, 900 µl of methanol and 100 µl of 2 mM FeCl_2_·2H_2_O was added to it and nurtured for 5 min. 400 µl of 5 mM ferrozine was added to initiate the reaction. The whole reaction mixture was further incubated for 10 min and then submitted to spectrophotometry at 562 nm by using EDTA as standard. The chelating power was determined by Eq. 1.

#### β-Carotene bleaching assay

β-Carotene bleaching activity of the plant was assessed by following the methodology suggested by Elzaawely et al. [51]. The format of this protocol was that a mixture of β-carotene was made by adding 2 mg of it with that of 10 ml chloroform followed by mixing 200 mg of Tween 80 and 20 mg of linoleic acid. The chloroform was evaporated out of the mixture by the help of vacuum and then 50 ml of distilled water was added to it, vigorously vortexed to get a uniform emulsion of β-carotene linoleate. Then 250 µl of freshly prepared emulsion was added to 30 µl of each plant sample and optical density was measured at time 0 h at wavelength 470 nm. The reaction mixture was kept at 45 °C for 2 h and the final optical density was measured again. Catechin served as standard in this assay. Inhibition of β-carotene was detected by slight alteration in the formula used by Mallet et al. [52]

$$ \% {\text{ inhibition }} = \, \left[ {\left( {{\text{A}}_{{{\text{A }}( 1 20)}} {-}{\text{ A}}_{{{\text{C }}( 1 20)}} } \right) \, /\left( {{\text{A}}_{{{\text{C }}(0)}} {-}{\text{ A}}_{{{\text{A }}( 1 20)}} } \right)} \right] \times 100 $$

where A_A (120)_ is the antioxidant absorbance at t = 120 min, A_C (120)_ is the control absorbance at t = 120 min, and A_C (0)_ is the control absorbance at t = 0 min.

#### Reducing power assay

By the method of Landry et al. [53] reducing power activity was calculated. Plant extract 2 ml was mixed with 2 ml of 0.2 M phosphate buffer (pH 6.6) and 2 ml of potassium ferricyanide (10 mg/l) were mixed up and incubated at 50 °C for 20 min. Then 2 ml of trichloroacetic acid (TCA) (100 mg/l) was added to the solution. After this 2 ml of the above solution was picked up and diluted with 2 ml of pure H_2_O and 0.4 ml of FeCl_3_ (0.1%) in a test tube. Standard used in this assay was gallic acid. Optical density was measured after 10 min at 700 nm.

#### Phosphomolybdenum assay

The antioxidant capabilities of the plant sample was assured by phosphomolybdenum assay as per described in the methodology of Umamaheswari and Chatterjee [54]. Phosphomolybdenum reagent solution was prepared by mixing Na_3_PO_4_ (28 mM) and H_2_SO_4_ (0.6 M) with that of ammonium molybdate (4 mM). The reaction mixture is heated at 95 °C in water bath for 90 min taking a good care that it is fully covered with silver foil to avoid direct light exposure. After this heat treatment, the reaction mixture was cooled at room temperature for some time and submitted to spectrophotometric analysis at 765 nm. Ascorbic acid serves as a standard in this assay.

### Statistical analysis

Experimental data results were conveyed in the form of mean ± standard deviation (SD) having triplicate analysis. The data was recognized and investigated by using the computerized GraphPad Prism (5.0) software to calculate the IC_50_ values. Statistix software 8.1 was used for further statistical analysis followed by applying ANOVA (One-way analysis variance) for the calculation of differences among various groups. The data is given as Additional file 1.

## Conclusion

The present study indicated the presence of higher polyphenol content and rich phytochemical assortment of *B. populneus* might be the key players in scavenging of oxidative stress inducing species. The result also suggests that the presence of various chemicals in this plant did not assort according to the polarity of solvents used in this study. This might led to the non-directional distribution and accumulation of chemicals in terms of yield.

## Additional file

**Additional file 1.** Phytochemical and antioxidant assays data. The data include the DPPH, Iron chelation, nitric oxide, hydroxyl, B-carotene, phenolics and flavonoids, phosphomolybdenum and reducing power assays sub files. It include all the data generated and analyzed for this study.

## Abbreviations

BPM: *B. populneus* methanol extract
BPH: *B. populneus n*-hexane fraction
BPC: *B. populneus* chloroform fraction
BPE: *B. populneus* ethyl acetate fraction
BPB: *B. populneus* butanol fraction
BPA: *B. populneus* aqueous fraction
TFC: total flavonoid content
TPC: total phenolic content
